# Quorum sensing modulates colony morphology through alkyl quinolones in *Pseudomonas aeruginosa*

**DOI:** 10.1186/1471-2180-12-30

**Published:** 2012-03-09

**Authors:** Rashmi Gupta, Martin Schuster

**Affiliations:** 1 Department of Microbiology, Oregon State University, Corvallis, OR 97331, USA

**Keywords:** Quorum sensing, *Pseudomonas aeruginosa*, Colony, Alkylquinolone, Acyl-homoserine lactone, Exopolysaccharide, Biofilm

## Abstract

**Background:**

Acyl-homoserine lactone (acyl-HSL) and alkyl quinolone (AQ) based quorum-sensing (QS) systems are important for *Pseudomonas aeruginosa *virulence and biofilm formation. The effect of QS on biofilm formation is influenced by various genetic and environmental factors. Here, we used a colony biofilm assay to study the effect of the central acyl-HSL QS regulator, LasR, on biofilm formation and structure in the representative clinical *P*. *aeruginosa *isolate ZK2870.

**Results:**

A *lasR *mutant exhibited wrinkled colony morphology at 37°C in contrast to the smooth colony morphology of the wild-type. Mutational analysis indicated that wrinkling of the *lasR *mutant is dependent on *pel*, encoding a biofilm matrix exopolysaccharide. Suppressor mutagenesis and complementation analysis implicated the AQ signaling pathway as the link between *las *QS and colony morphology. In this pathway, genes *pqsA-D *are involved in the synthesis of 4-hydroxyalkyl quinolines ("Series A congeners"), which are converted to 3,4-dihydroxyalkyl quinolines ("Series B congeners", including the well-characterized *Pseudomonas *Quinolone Signal, PQS) by the product of the LasR-dependent *pqsH *gene. Measurement of AQ in the wild-type, the *lasR pqsA*::Tn suppressor mutant as well as the defined *lasR*, *pqsH*, and *lasR pqsH *mutants showed a correlation between 4-hydroxyalkyl quinoline levels and the degree of colony wrinkling. Most importantly, the *lasR pqsH *double mutant displayed wrinkly morphology without producing any 3,4-dihydroxyalkyl quinolines. Constitutive expression of *pqsA-D *genes in a *lasR pqsR*::Tnmutant showed that colony wrinkling does not require the AQ receptor PqsR.

**Conclusions:**

Taken together, these results indicate that the *las *QS system represses Pel and modulates colony morphology through a 4-hydroxyalkyl quinoline in a PqsR-independent manner, ascribing a novel function to an AQ other than PQS in *P. aeruginosa*.

## Background

*Pseudomonas aeruginosa *is an important opportunistic human pathogen. It is known for its ability to inhabit diverse habitats ranging from soil to immunocompromised individuals [1]. In these environments, it can adopt either a planktonic or a surface-associated biofilm lifestyle. Biofilms, structured surface-associated microbial communities, are of considerable interest as they constitute an important survival strategy in infections [2]. *P. aeruginosa *forms different types of biofilms depending on the environment. In static liquid culture it forms pellicles at the air-liquid interface, under flow it can form solid surface-associated (SSA) biofilms and on solid agar medium it forms colonies [3]. Colonial growth is an easy and commonly used assay to study development of multicellular structures like biofilms [4-6].

**Figure 1:**
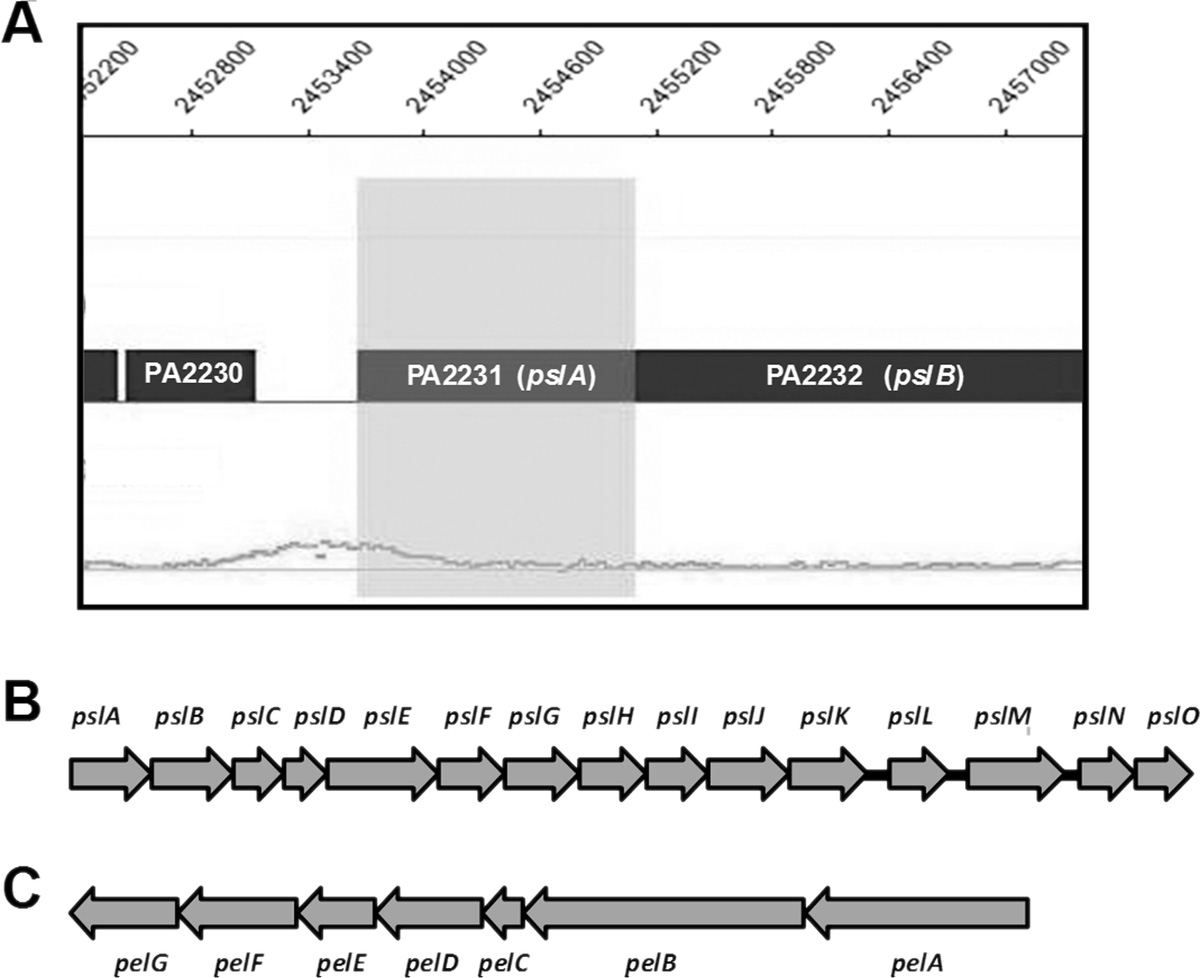
**Putative link between LasR and Psl control in *P. aeruginosa *PAO1**. **A**. CHIP-chip analysis performed with LasR-specific antibodies [8]. The signal peak near the bottom left corner of the panel indicates enrichment of *psl *promoter fragments and the vertical light grey bar represents the *pslA *gene (PA2231). The data were visualized using SignalMap (Nimblegen). **B**. *psl *EPS locus. **C**. *pel *EPS locus.

Biofilms are encased in a matrix composed of exopolysaccharide (EPS), but also extracellular DNA (eDNA), proteins, RNA and ions [7]. There are two main EPS in non-mucoid *P*. *aeruginosa*, Pel (encoded by *pelA-G*) and Psl (encoded by *pslA-O*) (Figure 1) [9-11]. Pel is glucose rich whereas Psl is galactose and mannose rich [11-13]. *P. aeruginosa *strain PA14 only contains *pel *while strains PAO1 and ZK2870 contain both *pel *and *psl *[11,12]. All of these strains are clinical isolates that differ in their aggregative behavior. While strains PA14 and PAO1 are the most commonly used laboratory strains, strain ZK2870 with its autoaggregative phenotype is believed to be the most representative among clinical strains [12].

Quorum sensing (QS) is a cell density-dependent mechanism of bacterial communication that coordinates other group behaviors. *P. aeruginosa *has two complete acyl-homoserine lactone (acyl-HSL)-based QS systems, *las *and *rhl *[14,15]. They consist of the transcriptional regulators LasR and RhlR and the signal synthases, LasI and RhlI, respectively. LasI and RhlI catalyze the synthesis of N-3-oxododecanoyl-homoserine lactone (3OC12-HSL) and N-butryl-homoserine lactone (C4-HSL), which bind and activate their cognate transcriptional regulators LasR and RhlR, respectively. Both systems are arranged in a hierarchical manner with the *las *system controlling the *rhl *system [16,17]. A third QS system in *P. aeruginosa*, *pqs*, is based on alkyl quinolones (AQ) [18,19]. This system connects both the *las *and *rhl *QS systems. It includes the transcriptional regulator PqsR (MvfR), which positively regulates the expression of the *pqsA-E *operon. PqsA-D enzymes are involved in the synthesis of 4-hydroxyalkyl quinolines (named Series A congeners by Deziel *et al.*) [20]. This class of compounds is converted to 3, 4 dihydroxyquinolines (Series B congeners) by a monoxygenase encoded by the *pqsH *gene [20]. The most prominent Series A congeners are 4-hydroxy-2-heptyl quinoline (HHQ) and 4-hydroxy-2-nonyl quinoline (HNQ), and the most prominent Series B congener is 3,4-dihydroxy-2-heptyl quinoline (PQS), due to their established roles as cell-cell signaling molecules. HHQ/HNQ and PQS bind PqsR with low and high affinity, respectively, and are capable of activating the protein [21-23]. LasR positively regulates AQ production by upregulating *pqsR *[22] and *pqsH *[20,24] transcription, although under certain culture conditions, AQ can also be produced in the absence of a functional *las *system [25]. The *rhl *system, in turn, represses *pqsR *and *pqsA-E *expression [22,26,27]. The AQ biosynthetic enzymes enable *P. aeruginosa *to produce more than 50 distinct AQ molecules [20,28]. Together, the three QS systems, *las*, *rhl*, and *pqs*, regulate > 5% of the *P. aeruginosa *genome [29-32].

Several studies have investigated the contribution of each QS system to biofilm formation. A functional *las *system is required for formation of highly structured SSA biofilm communities in *P. aeruginosa *PAO1 [33]. The *las *system influences biofilm matrix formation and activation of *pel *EPS [6]. In another study, the *las *system was shown to indirectly inhibit *pel *expression through weak activation of the tyrosine phosphatase TpbA [34]. The *rhl *QS system contributes to maintenance of biofilm architecture through production of rhamnolipid surfactants [35]. The *pqs *system in turn is implicated in autolysis [36] and maintaining biofilm integrity as a consequence of eDNA release [37]. In addition, the contribution of QS to biofilm formation is modulated by environmental factors such as nutritional cues [38]. Taken together, the role of QS in biofilm formation is multifactorial.

Our recent work suggested yet another connection between QS and EPS production. We showed by chromatin immunoprecipitation-microarray analysis (CHIP-chip) and electrophoretic mobility shift assay that LasR binds to the putative promoter region of the Psl EPS operon [8] (Figure 1). This finding led us to investigate in more detail how *lasR *mutation affects EPS production and colony biofilm formation. A *lasR *mutant of *P*. *aeruginosa *strain ZK2870 exhibited a pronounced wrinkled colony morphology at 37°C suggesting a possible link between *las *QS and *psl *expression. However, we found that the wrinkled phenotype is *pel *rather than *psl*-dependent. Subsequent suppressor mutagenesis in the *lasR *mutant background implicated the involvement of the *pqs *pathway. Phenotypic analysis and quantitation of AQ levels by thin-layer chromatography (TLC) of several QS mutants revealed that a Series A congener, likely other than HHQ or HNQ, modulates the structural organization of a colony. This study broadens the functional significance of AQ production by *P. aeruginosa*.

Our recent work suggested yet another connection between QS and EPS production. We showed by chromatin immunoprecipitation-microarray analysis (CHIP-chip) and electrophoretic mobility shift assay that LasR binds to the putative promoter region of the Psl EPS operon [8] (Figure 1). This finding led us to investigate in more detail how *lasR *mutation affects EPS production and colony biofilm formation. A *lasR *mutant of *P*. *aeruginosa *strain ZK2870 exhibited a pronounced wrinkled colony morphology at 37°C suggesting a possible link between *las *QS and *psl *expression. However, we found that the wrinkled phenotype is *pel *rather than *psl*-dependent. Subsequent suppressor mutagenesis in the *lasR *mutant background implicated the involvement of the *pqs *pathway. Phenotypic analysis and quantitation of AQ levels by thin-layer chromatography (TLC) of several QS mutants revealed that a Series A congener, likely other than HHQ or HNQ modulates the structural organization of a colony. This study broadens the functional significance of AQ production by *P. aeruginosa*.

## Methods

### Bacterial strains and growth conditions

Strains and plasmids are listed in Table 1. We used three strains of *P. aeruginosa *in this study, namely the widely used clinical isolates PAO1 and PA14, and the more recent clinical isolate ZK2870 (herein abbreviated as ZK) [12]. Bacterial cultures were grown at 22°C and 37°C as specified. Lennox broth (LB) [8] or tryptone broth [12] were used for routine culturing. Tryptic soy broth (TSB) was used for flow-cell biofilm assays. Where appropriate, antibiotics were added to the growth media as follows: Tetracycline and gentamicin, 100 μg/ml for *P. aeruginosa *and 10 μg/ml for *Escherichia coli*; carbenicillin, 200 μg/ml for *P. aeruginosa*; ampicillin, 100 μg/ml for *E. coli*.

**Table 1:** Strains and plasmids

Strain or plasmid Strain	Relevant property	Reference
***P. aeruginosa***		
PA14	Wild-type	[39]
PAO1	Wild-type	[40]
ZK2870	Wild-type	[12]
PAO1 *lasR*	Markerless *lasR *mutant derived from PAO1	[41]
PA14 *lasR*	Tn*phoA lasR *mutant derived from PA14	[42]
ZK *lasR*	Markerless in-frame *lasR *deletion in ZK2870	This study
ZK *pelA*	Markerless *pelA *deletion in ZK2870	[11]
ZK *pslD*	Markerless *pslD *deletion in ZK2870	[11]
ZK *lasI*	Markerless *lasI *deletion in ZK2870	This study
ZK *pelA lasR*	Markerless *lasR *deletion in a *pelA *mutant of ZK2870	This study
ZK *pslD lasR*	Markerless *lasR *deletion in a *pslD *mutant of ZK2870	This study
ZK *pqsH*	Markerless *pqsH *deletion in ZK2870	This study
ZK *tpbA*	Markerless *pqsH *deletion in ZK2870	This study
ZK *lasR*	*pqsA *suppressor mutation in a *lasR *mutant of ZK2870	This study
*pqsA*::Tn		
ZK *lasR*	*pqsR *suppressor mutation in a *lasR *mutant of ZK2870	This study
*pqsR*::Tn		
***E. coli***		
DH5α	F^- ^φ80d*lacZ*ΔM15 Δ (*lacZYA-argF*) U169 *deoR recA1 endA1 hsdR17*(r_K_^- ^m_K_^+^) *phoA supE44 *λ^-^*thi*-*1 gyrA96 relA1*	Invitrogen
SM10	Mobilizing strain, RP4 *tra *genes integrated in the chromosome, *thi-1 thr leu tonA lacY supE recA*::RP4-2-Tc::Mu Km^r^	[43]
S17-1/λpir	Tp^R ^Sm^R ^*recA, thi, pro, hsdR*-M^+^RP4: 2-Tc:Mu: Km Tn7 λpir	[43]
**Plasmid**		
mini-CTX-*lacZ*	Chromosomal integration vector, Tc^R^	[44]
pEX18 Tc	Allelic exchange suicide vector, Tc^R^	[45]
pEX18.Δ*lasR*	Suicide vector with *lasR *in-frame deletion	[41]
pEX18.Δ*lasI*	Suicide vector with *lasI *in-frame deletion	[41]
pEX18.Δ*tpbA*	Suicide vector containing *tpbA *in-frame deletion	This study
pLM1	Tn5 delivery vector, Gm^R^	[46]
pLG10	*pqsA-E *operon cloned in pUCP18, Ap^R^	[24]
pRG10	*pqsA-D *operon cloned under control of P*_lac _*of pUCP18, Ap^R^	This study
pRG11	Promoter region of *pel *cloned in mini-CTX-*lacZ *vector	This study
pUCP18	Parent vector of pLG10, Ap^R^	[47]

### Strain and plasmid constructions

Deletion mutants were constructed using the strategy of Hoang *et al. *[45]. ZK *lasR *and *lasI *mutants were generated by introducing the previously constructed allelic exchange plasmids pEX18.Δ*lasR *and pEX18.Δ*lasI*, respectively [41], into the parent strain and selecting on LB agar containing nalidixic acid (20 μg/ml) and tetracycline. Double cross-over recombinants were further selected on LB plates supplemented with 5% sucrose [45]. The *pqsH *and *tbpA *in-frame deletions were constructed using SOE-PCR [48]. The respective primers are listed in Additional file 1: Table S1. The deletion constructs obtained from SOE-PCR were digested with the appropriate restriction enzymes (see Additional file 1: Table S1) and ligated into equally digested pEX8 [45]. The resulting constructs pEX18.Δ*pqsH *and pEX18.Δ*tpbA *were transformed into *E. coli *SM10. Mating with *P. aeruginosa *ZK and appropriate selection as discussed above yielded *pqsH *and *tpbA *deletion mutants. The *pelA lasR *and *pslD lasR *double mutants were constructed by generating an in-frame *lasR *deletion (as described above) in *pelA *and *pslD *mutant backgrounds, respectively. A *lasR pqsH *double mutant was constructed by *pqsH *deletion in a *lasR *mutant background. Proper construction of deletion mutants was confirmed by PCR amplification of chromosomal DNA. The plasmid pRG10 was constructed by amplifying a 5.5 kb region containing the *pqsA*-*D *genes using appropriate primers (see Additional file 1: Table S1) and cloning between the *Pst*I and *Hin*dIII restriction sites of the pUCP18 vector [47].

### Colony biofilm assay

Bacterial cultures were grown overnight in LB at 37°C. The overnight culture was diluted to an optical density (OD_600_) of 0.0025 in tryptone broth and 10 μl of the diluted culture was spotted onto Congo red plates [12]. The Congo red medium contained tryptone (10 g/l), granulated agar (0.5%), Congo red (40 mg/l), and Coomassie brilliant blue R 250 (20 mg/l). The plates were wrapped with aluminum foil and incubated at 37°C for 3-5 days. For bacterial strains containing plasmid pLG10 or pRG10, carbenicillin was added to the medium.

### Chemical supplementation

3OC12-HSL was added to Congo red plates buffered with 50 μM 3-(N-morpholino) propanesulfonic acid (MOPS) at a final concentration of 10 μM. Pyocyanin was added to Congo red plates at a final concentration of 50 μM. HHQ (a gift from M. whitelely, University of Texas) and HNQ (a gift from P. Williams, University of Nottingham) were added to MOPS-buffered Congo red plates at a final concentration of 50 μM or directly to the bacterial inoculum at final concentrations of 20, 100 and 500 μM. The respective solvents ethyl acetate, dimethyl sulfoxide (DMSO), and methanol were used as controls.

### *Pel'-lacZ-*reporter construction and β-galactosidase measurements

A 555 bp promoter region of the *pel *operon was amplified from the ZK strain using the primers listed in Additional file 1: Table S1 and cloned upstream of the *lacZ *gene in the integration vector mini-CTX-*lacZ *[44]. The resulting plasmid pRG11 was then inserted into the chromosome of the wild-type and the *lasR *mutant as described [44]. As a control, the mini-CTX-*lacZ *parent vector was also integrated into the genome. The colonies of the ZK wild-type and the *lasR *mutant grown on Congo red plates at 37°C were used to measure β-galactosidase levels. A colony was cut out on the 3^rd^, 4^th^, and the 5^th ^day and suspended in 2 ml of 50 mM phosphate buffer, pH 7.4, in a 15 ml conical tube. Cells were lysed by sonication. The total protein was estimated by Bradford assay [49]. The sonicated sample was centrifuged at 4°C for 30 min. The resulting supernatant was used to measure β-galactosidase activity as described previously [50].

### Pellicle biofilm assay

Cultures were inoculated in tryptone broth at an OD_600 _of 0.0025 and incubated at 22°C and 37°C without shaking [11]. After 24, 48 and 72 h, pellicle formation was observed at the air-liquid interface.

### Microtiter plate biofilm assay

Biofilm formation in a microtiter format was assayed as described [11]. Overnight cultures of the wild-type and the *lasR *mutant grown in LB broth at 37°C were diluted 1:100 in tryptone broth. One hundred and fifty μl of the diluted culture was added to 96-well polystyrene microtiter plates (Cellstar-Greiner Bio-one) and incubated at 22°C and 37°C without shaking for 48 and 72 h. Microtiter plates were rinsed in running hot water. Adherent cells were then stained with 1% crystal violet for 20 min. The microtiter plate was again rinsed in running hot water. Ethanol was added to each dry well and the samples were allowed to stand for 20 min. Absorbance was measured at 590 nm.

### Flow-cell biofilm assay

Biofilms were grown at 37°C in flow chambers. The system was assembled as described [33,51]. The cultures for inoculation were prepared from mid-exponential phase (OD_600 _of 0.4-0.8) TSB cultures grown at 37°C. The cultures were diluted to an OD_600 _of 0.05 in 1:100 diluted TSB medium and injected into the flow cell. Flow was initiated after 1 h. The diluted TSB was supplied at a flow rate of 180 μl/min using a peristaltic pump (Watson Marlow 205S). Images were taken when biofilms had matured (day 3) with an inverted Zeiss LSM Zeta 510 confocal laser scanning microscope (CLSM) using a 63X oil immersion lens. The manufacturer's software and Adobe Photoshop were used for image processing.

### Suppressor mutagenesis

For transposon mutagenesis, biparental matings were set up between the *E. coli *donor (S17-1-λpir/pLM1) and the *P. aeruginosa *recipient strain (ZK *lasR *mutant) as described [52]. The suicide plasmid pLM1 carries a miniTn5 transposon. The transposon insertion mutants were selected on LB agar plates containing gentamicin (30 μg/ml) and nalidixic acid (20 μg/ml). Colonies were picked manually and patched onto rectangular LB plates containing gentamicin (30 μg/ml) in a 96-well format. Plates were incubated at 37°C for one day and then replica-plated onto rectangular Congo red plates using a 96-well-pin replicator. The ZK wild-type and the *lasR *mutant were included as controls. These plates were incubated for 3- 5 days at 37°C. Candidate revertants exhibiting a smooth colony morphology identical to the wild-type were streaked for isolated colonies and subjected to a second screen. This screen involved performing the original colony biofilm assay as described earlier. Mutants which again showed a smooth phenotype were considered to be true revertants.

### Mapping of transposon insertions

Genomic DNA was isolated from the selected transposon mutants (Qiagen PUREGENE kit) and was digested with *Nco*I. The transposon does not contain an *Nco*I restriction site and has an R6K origin of replication. The digested DNA was self-ligated with T4 DNA ligase (New England Biolabs) and electroporated into chemically competent *E. coli *S17-1/λpir [43]. Plasmid DNA was isolated from gentamicin-resistant colonies and was sequenced using the Tn5 specific primer tnpRL17-1 [53]. Transposon insertions were mapped by comparing sequences to a *Pseudomonas *protein database using BlastX.

### Overexpression of *pqsA-E*

The appropriate strains were transformed with plasmid pLG10 [24] and pRG10 carrying the *pqsA-E *operon and *pqsA-D *operon under the control of native and constitutive promoters, respectively, or with pUCP18 [47], the parent vector from which pLG10 and pRG10 were derived.

### Thin-layer chromatography (TLC)

Samples for TLC analysis were prepared from 3-5 day-old colonies. Two colonies of each strain grown on the same plate were cut out from the agar with minimum possible agar contamination. One colony was used for total protein estimation and the other for AQ extraction. Total protein was estimated by Bradford assay [49] as described earlier for β-galactosidase measurements. For AQ extraction, a colony was harvested and suspended in 5 ml methanol, homogenized with a tissue tearor, and allowed to stand for 10 min. The suspension was centrifuged for 30 min at 4000 r.p.m. at 4°C. The supernatant was filtered through a 0.2 μM syringe filter and the filtrate was collected in glass vials prewashed with acetone. The samples were then air-dried, reconstituted in 500 μl of methanol and transferred to 2 ml glass vials. They were again air-dried and finally reconstituted in 100 μl of methanol. TLC plates were prepared and samples were run as described [23]. Five μl of the sample (normalized to total protein), 2 μl of the standards-PQS (5 and 10 mM), and HHQ (2.5 and 5 mM,) were used. AQ levels were estimated in the wild-type and the *lasR *mutant by densitometric analysis of relative spot intensities using Imagequant TL software (GE Healthcare) from two independent experiments.

## Results and discussion

### A ZK *lasR *mutant forms wrinkly colonies

**Figure 2:**
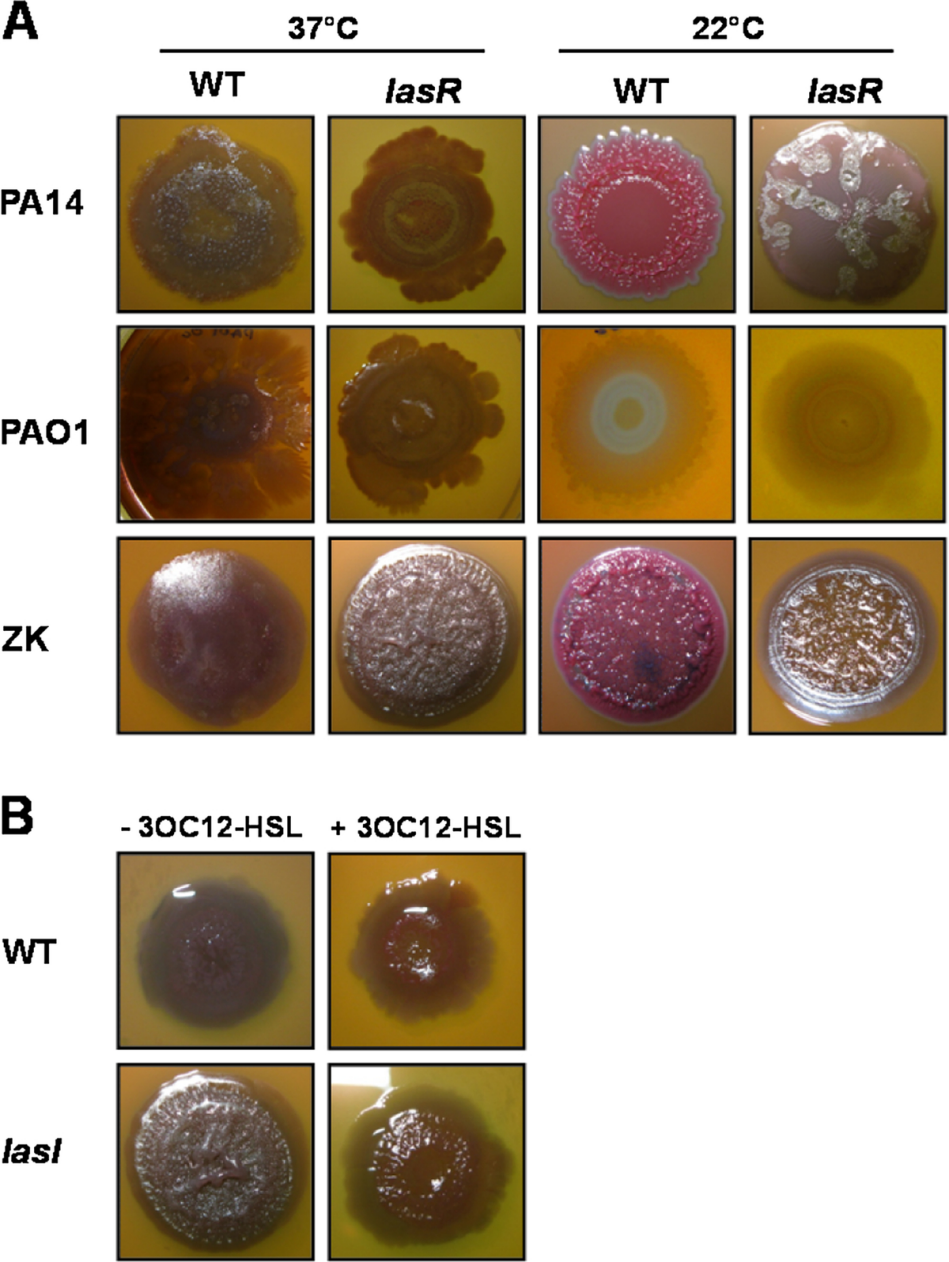
**Effect of *las *mutation on colony wrinkling**. **A**. Colony morphology of wild-type (WT) and *lasR *mutant *P. aeruginosa *strains PA14, PAO1 and ZK after 5 days of growth at the indicated temperature. **B**. Colony morphology of the ZK wild-type (WT) and *lasI *mutant in the presence and absence of 10 μM 3OC12-HSL after 5 days at 37°C.

We investigated the effect of a *lasR *mutation on colony morphology as an indicator of matrix production [6,12]. A wrinkled colony phenotype is generally associated with increased EPS production and biofilm formation. Our agar medium also contained Congo-red, which may stain colonies overproducing EPS [54], but is not always a reliable indicator, especially at 37°C [5]. We therefore focused on colony wrinkling (rugosity). We grew the wild-type and *lasR *mutants of three *P. aeruginosa *strains, namely widely used strains PAO1 and PA14, and the autoaggregative strain ZK2870 [12], on agar plates for 5 days at 37°C and at 22°C. Growth conditions are identical to those previously used to investigate EPS-dependent colony morphology [6,12]. We did not observe any significant differences in rugosity between the PAO1 wild-type and *lasR *mutant strains at either temperature (Figure 2A). However, the colonies of the wild-type and the *lasR *mutant of strains PA14 and ZK showed striking differences. A PA14 *lasR *mutant formed a flat, smooth colony as compared to the wrinkled wild-type phenotype at 22°C (Figure 2A). On the contrary, a ZK *lasR *mutant formed a distinctive wrinkled colony at 37°C while the wild-type formed a smooth colony (Figure 2A). At room temperature, the morphological difference between the wild-type and the ZK *lasR *mutant was not as pronounced. A positive regulatory link between *las *QS, *pel *transcription and colony morphology has already been described in strain PA14, which only carries Pel EPS [6]. The apparently reverse relationship between *las *QS and colony morphology at 37°C in strain ZK, which harbors both Pel and Psl, was intriguing to us and is the focus of this study.

To confirm that the observed phenotype is generally dependent on a non-functional *las *system, we also constructed a ZK *lasI *in-frame deletion mutant. A ZK *lasI *mutant showed a well defined wrinkled colony like the *lasR *mutant at 37°C (Figure 2B). Supplementation of the *lasI *mutation with exogenous 3OC12-HSL signal virtually restored the smooth wild-type phenotype (Figure 2B). This confirms that the *las *system is responsible for the wrinkled colony phenotype. We used the ZK *lasR *mutant for further study.

### Genetic analysis indicates involvement of *pel *rather than *psl*

**Figure 3:**
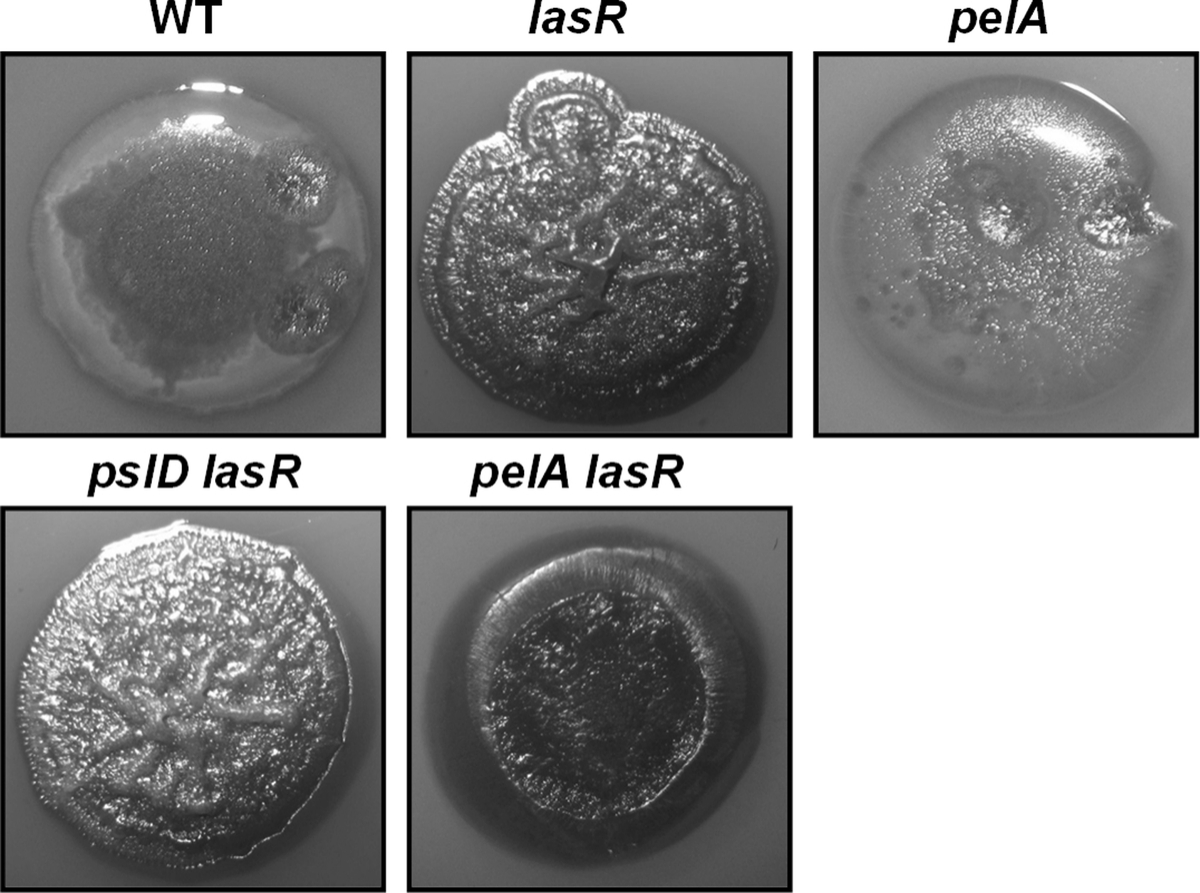
**Genetic analysis of *pel *and *psl *involvement**. Colony morphology of the ZK wild-type (WT), *lasR *mutant, *pelA *mutant, *pelA lasR *and *pslD lasR *double mutants after 5 days of growth at 37°C.

We performed mutational analysis to investigate whether Pel or Psl EPS might cause wrinkling of the *lasR *mutant. We constructed *pelA lasR *and *pslD lasR *double mutants and compared their colony morphology to that of the *lasR *mutant and the wild-type parent. A *pelA lasR *double mutant showed a nearly smooth colony phenotype while the *pslD lasR *mutant showed a wrinkled phenotype like the *lasR *mutant (Figure 3). We evaluated the contribution of *pel *alone by comparing the colony morphology of a *pelA *mutant to the wild-type. The *pelA *colony phenotype was indistinguishable to that of the wild-type. The partial loss of wrinkles in a *pelA lasR *double mutant therefore indicates inhibition of Pel by LasR.

To determine whether inhibition is at the transcriptional level, we measured *pelA *transcription in the wild-type and the *lasR *mutant using a *pelA**'**-lacZ *transcriptional fusion inserted at a neutral chromosomal site. We harvested colonies after 3, 4 and 5 days, because a ZK *lasR *mutant begins to show wrinkling at day 3. We found no difference in *pelA *transcription in the wild-type and the *lasR *mutant (data not shown). This indicates that *pel *regulation is at the posttranscriptional level. We attempted to investigate this possibility by quantifying EPS; however, we were unable to perform an EPS composition and linkage analysis because of insufficient amounts of purified EPS extracted from colonies required for such analysis.

### Investigation of other factors associated with *pel *and the wrinkled colony phenotype

**Figure 4:**
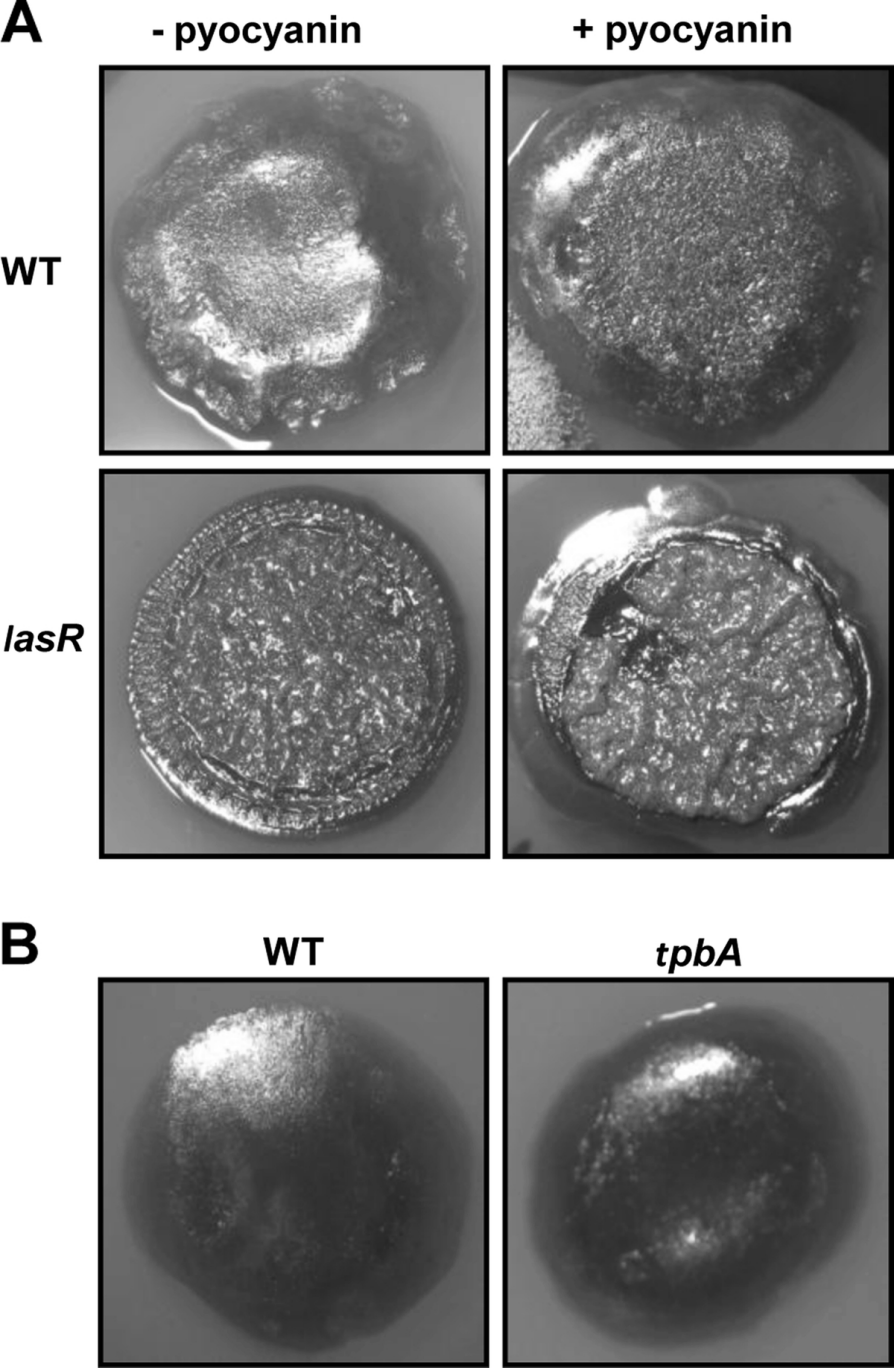
**Role of pyocyanin and *tpbA *in the wrinkled colony phenotype**. **A**. Colony morphology of the ZK wild-type (WT) and the *lasR *mutant with and without 50 μM pyocyanin. **B**. Colony morphology of the ZK wild-type (WT) and the *tpbA *mutant. Colonies were grown for 3 days at 37°C.

We investigated the role of phenazines and of the tyrosine phosphatase TpbA in the observed wrinkled phenotype of a ZK *lasR *mutant as both modulate structural organization of *P*. *aeruginosa *PA14 colony biofilms [34,55]. We examined the relationship between phenazine deficiency and the wrinkled phenotype through addition of pyocyanin to the agar medium. Pyocyanin supplementation did not result in loss of wrinkles in the *lasR *mutant (Figure 4A). Inhibition of TpbA in strain PA14 has been shown to enhance *pel *expression at 37°C, resulting in a wrinkled colony phenotype [34]. We therefore constructed a *tpbA *mutant in the ZK background and examined colony morphology. The *tpbA *mutant remained as smooth as the wild-type (Figure 4B). These results indicate neither pyocyanin nor TpbA are responsible for the wrinkled phenotype of the ZK *lasR *mutant.

### Hydrated *lasR *mutant biofilms do not show altered architecture

**Figure 5:**
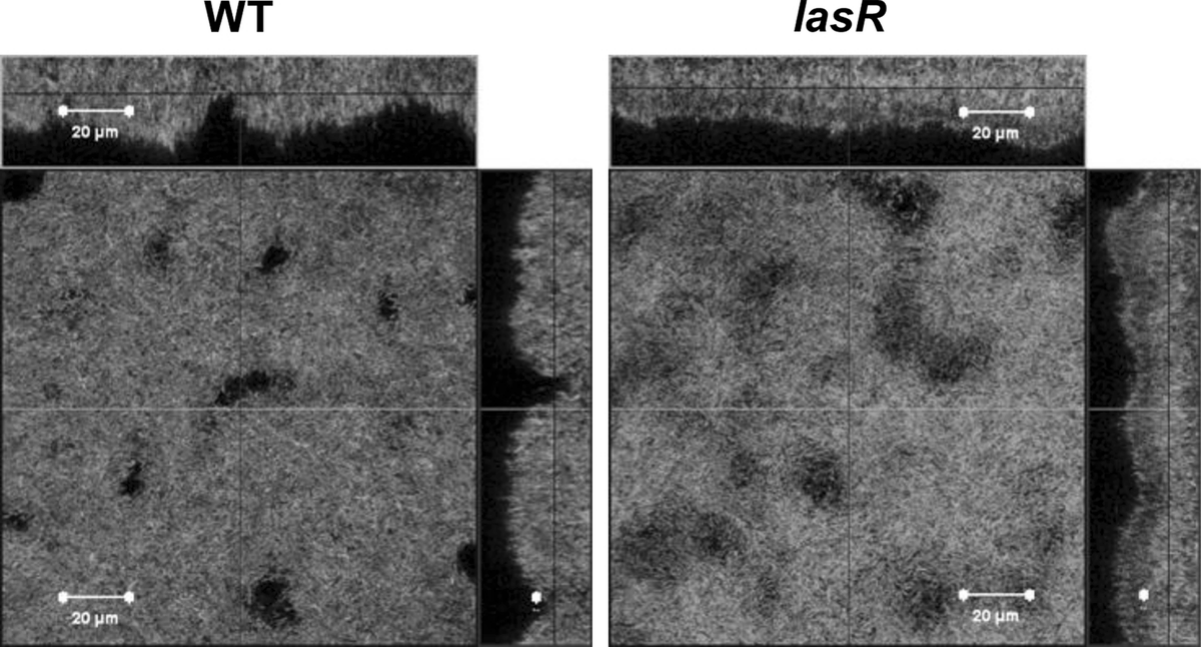
**Flow-cell biofilms**. CLSM images of flow-cell grown biofilms of the ZK wild-type (WT) and the *lasR *mutant at 37°C after 3 days. The large panel shows the horizontal cross-section and the small panel shows the vertical cross-section of the biofilm. The lines in the panels indicate the planes of the cross-sections.

The involvement of *pel *in the wrinkled colony morphology of the ZK *lasR *mutant suggested that it might exhibit generally altered biofilm architecture. We investigated pellicle formation of standing cultures as well as biofilm formation in microtiter plates and flow-cells. Flow-cell biofilms of the wild-type and the *lasR *mutant after 3 days of growth are shown in Figure 5. Neither assay revealed any differences between the two strains. This is consistent with recent results by Colvin *et al.*, who also found no defect in attachment or biofilm development for a *pel *mutant of strain PAO1 [56]. There is a difference in the degree of hydration in the three biofilm assays we employed. Submerged flow-cell biofilms are fully saturated and hydrated, pellicles and microtiter plate biofilms that form at the air-liquid interface are somewhat less hydrated, whereas colonies on agar are the least hydrated [57]. It is possible that the observed phenotype only manifests under conditions of low hydration.

### Suppressor mutagenesis implicates the *pqs *pathway

**Figure 6:**
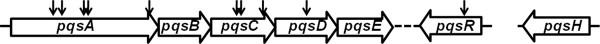
**The *pqs *locus and transposon insertions in associated suppressor mutants**. Horizontal arrows represent the genes of the *pqsA-E *operon, the *pqsR *transcriptional regulatory gene, and the *pqsH *gene. Small vertical arrows indicate the location of the respective transposon insertions.

Transposon mutagenesis was performed in the ZK *lasR *mutant background to identify the regulatory link between the *las *QS system and colony morphology. Around 10,000 mutants were screened for reversion to a smooth phenotype. We identified 38 mutants, and mapped transposon insertions in 25 (Additional file 2: Table S2). We found 9 transposon insertions in the *pqsA-D *genes of the AQ biosynthesis operon and one insertion in the gene encoding the transcriptional regulator PqsR that activates *pqsA-E *expression (Figure 6). Given the large fraction of hits (10 out of 25 or 40%), the role of the *pqs *operon was apparent even without mapping the remaining transposon mutants. We did not identify any insertions in *pqsH*, which promotes the conversion of Series A (4-hydroxyalkyl quinolines) to Series B (3,4 dihydroxyalkyl quinolines) congeners nor in *pqsE*, which encodes a putative global regulator [20,58]. Surprisingly, we also did not identify a transposon insertion in the *pel *operon, although our data in Figure 3 show that the *lasR pel *mutant forms a smooth colony. We found that this mutant displayed very slight wrinkling under the conditions employed for the high throughput screen, in which our primary focus was on the identification of the most obvious smooth revertants.

**Figure 7:**
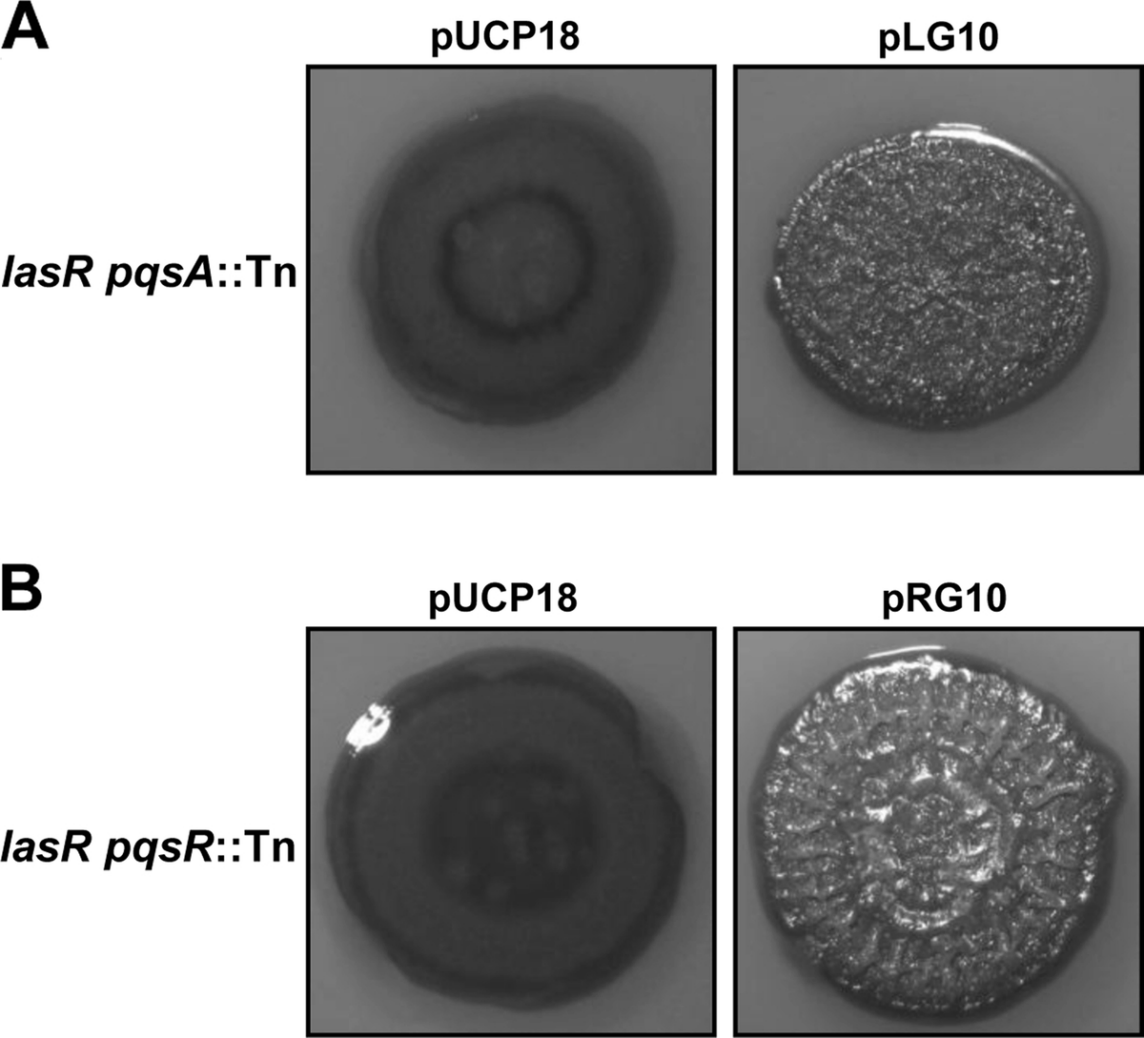
**Effect of ectopic *pqs *operon expression on colony morphology**. **A**. Colony morphology of the ZK *lasR pqsA*::Tn suppressor mutant with plasmid pLG10 expressing *pqsA-E *from the native promoter or control plasmid pUCP18 after 3 days at 37°C **B**. Colony morphology of the *lasR pqsR*::Tn suppressor mutant with plasmid pRG10 expressing *pqsA-D *from a constitutive *lac *promoter or control plasmid pUCP18 after 4 days at 37°C.

The wrinkly phenotype of the *lasR pqsA*::Tn suppressor mutant could be restored by introducing plasmid pLG10 [24], which expresses the *pqsA-E *operon from its native promoter (Figure 7A). This verifies that the products of this operon are indeed responsible for the wrinkled phenotype of the *lasR pqsA*:Tn mutant. To investigate whether *pqsA-D *dependent wrinkling of the *lasR *mutant is through PqsR, we introduced plasmid pRG10 into the *lasR pqsR*:Tn mutant. This plasmid constitutively expresses the *pqsA-D *operon from a *lac *promoter. The *lasR pqsR*:Tn mutant colony was as wrinkly as that of the *lasR *mutant indicating that this phenotype is independent of PqsR (Figure 7B).

### A Series A AQ congener causes the wrinkled phenotype

**Figure 8:**
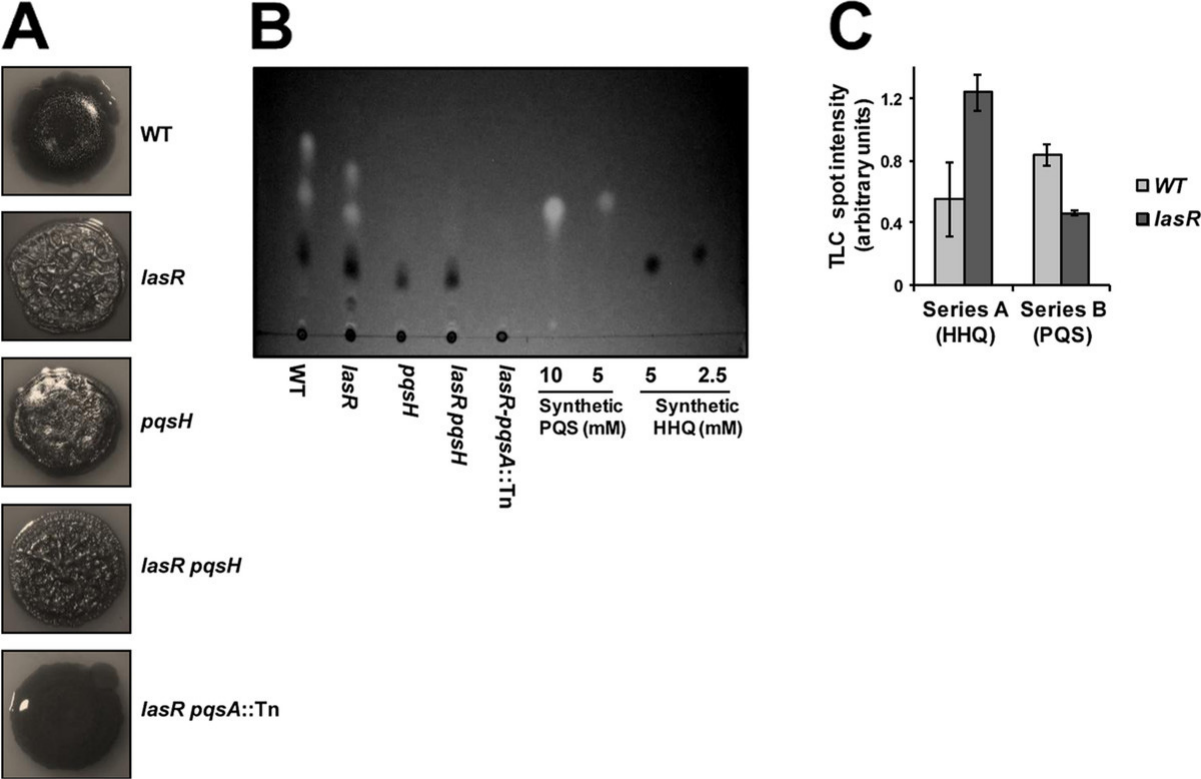
**Colony morphology and AQ production of various QS mutants**. **A**. Colony morphology of the ZK wild-type (WT), *lasR*, *pqsH*, *lasR pqsH *double mutant, and *lasR pqsA*::Tn suppressor mutant after 5 days at 37°C. **B**. TLC analysis of AQ production by the respective strains. Approximately 5 μl of each sample (normalized to total amount of protein) was loaded. Note that samples towards the center of the plate ran more slowly than those near the edges. HHQ and PQS, representing Series A and B congeners, respectively, were included as synthetic controls. **C**. Densitometric analysis of TLC spot intensities in the wild-type and the *lasR *mutant from two independent experiments.

The previous finding that *lasR *mutants overproduce Series A congeners [20,59] and the fact that we did not find any insertion in the *pqsH *gene indicate that Series A congeners rather than Series B congeners are responsible for the wrinkled phenotype. We therefore examined this notion further by correlating colony morphology and AQ production, as measured by TLC, in a number of mutant strains. TLC allowed us to distinguish between high-abundance Series A and B congeners. This assay was developed and has been optimized to detect PQS and HHQ, owing to their important roles in cell-cell signaling. Compounds within each series, especially C7 and C9 congeners, are not well separated, and low-abundance compounds may not be detectable [23]. We included the wild-type, the *lasR *mutant, and the *lasR pqsA*::Tn suppressor mutant in this analysis. In addition, we constructed a *pqsH *single mutant and a *lasR pqsH *double mutant in the ZK background. If a Series A congener caused wrinkling, then a *lasR pqsH *mutant should still be wrinkly, and a *pqsH *mutant would also be wrinkly if a Series A congener accumulates. Indeed, the degree of wrinkling generally correlated well with the amount of Series A congener produced, in the order *lasR-pqsA*::Tn < WT <*pqsH *<*lasR *and *lasR pqsH *(Figure 8A). The wrinkly *lasR *mutant and the *lasR pqsH *double mutant produce the most, while the smooth wild-type produces considerably less (Figure 8B). The fact that the wrinkly *lasR pqsH *mutant does not produce Series B congeners implies a role for a Series A congener. It is not clear why the *pqsH *single mutant does not overproduce Series A congeners as previously shown for strain PA14 [27]. It is possible that *pqsA-E *expression is reduced because indirect inhibition by the *las *system via the *rhl *system and the lack of the strong inducer PQS have a larger effect in the ZK background. Regardless, the partially wrinkly phenotype of the *pqsH *mutant indicates that in addition to absolute abundance, the ratio of Series A to B congeners may also be important. Densitometric analysis of wild-type and *lasR *mutant TLC spot intensities indeed shows that the Series A to Series B ratio is reciprocal in the two strains (Figure 8C).

Two Series A compounds, the PQS precursor HHQ and HNQ, have been shown to be overproduced in a *lasR *mutant [20]. To examine whether one of these compounds is responsible for the wrinkly morphology of the *lasR *mutant, we added them to the *lasR pqsA *suppressor mutant. Exogenous addition to the agar medium or directly to the bacterial inoculum did not result in any change in colony morphology (data not shown). It is possible that diffusible AQ compounds are unable to enter cells in sufficient quantity, or that another less well-characterized Series A congener is responsible for the observed phenotype. Because exogenous complementation with diffusible AQ has been successful in the past [60,61], we favor the latter.

## Conclusion

In this study, we investigated the effect of *las *QS on biofilm formation and structure using a colony biofilm approach. This work was motivated by our recent global position analysis of LasR, which showed that this regulator directly binds to the *psl *polysaccharide promoter [8] (Figure 1). While we were unable to demonstrate the significance of this finding in the present study, we established a novel connection between *las *QS and the other major *P*. *aeruginosa *EPS, Pel. In particular, we provide genetic evidence suggesting that the LasRI system represses Pel. We do not have any other independent evidence of this regulatory link as EPS composition analysis was unsuccessful. Las QS also only affected colonial morphology and did not affect biofilm formation in other relevant assays, including microtiter plate, pellicle, and flow-cell. It is conceivable that water availability (matric stress) is responsible for the conditionality of the observed phenotype.

It has previously been shown that LasRI induces Pel expression in strain PA14 at room temperature but not at 37°C [6]. This regulation is probably indirect and mediated via an unknown transcriptional regulator. Our finding that LasRI can also repress Pel expression in strain ZK at 37°C, a temperature relevant to infection, raises the intriguing possibility that QS may trigger dissolution of clinical biofilms. This would be analogous to other bacterial pathogens like *Vibrio cholerae *[62] and *Staphylococcus aureus *[63]. Results with the particular strain chosen, ZK2870, are significant, because the autoaggregative behavior of this strain under some environmental conditions appears most representative among clinical and environmental isolates of *P. aeruginosa *[12]. The observed differences in the colony phenotype of different *Pseudomonas *strains (Figure 2) might be attributed to the presence or absence of a particular EPS locus or regulatory variability in strains with identical EPS loci.

Our second major finding is that *las *QS mediates colony morphology via AQ signaling. Phenotypic analysis along with AQ signal quantitation by TLC suggested that a Series A congener is involved. PqsA-D produce at least 8 different compounds within this series [64]. Of these, HHQ and HNQ have been shown to accumulate in a PAO1 *lasR *mutant [20]. Other prominent AQs, 2,4-dihydroxyquinoline (DHQ) and 2-heptyl-4-hydroxyquinoline *N*-oxide (HQNO), that require some of the enzymes encoded by *pqsA-D*, but are not PqsH substrates, show reduced levels in a *lasR *mutant compared with the wild-type [20]. Our chemical supplementation experiments indicate that neither HHQ nor HNQ modulate wrinkling. This implies that one of the other less-well characterized Series A congeners have a role in this process, further expanding the various cellular functions of AQs in *P. aeruginosa*. A detailed investigation utilizing liquid chromatography/mass spectrometry along with chemical synthesis would be able to identify the compound in question. PqsE, a putative regulator encoded by the *pqsA-E *operon whose precise function is not known, is unlikely to have a role in AQ-mediated colony wrinkling, because *pqsA-D *expression in a *lasR pqsR *mutant that does not express *pqsE *was sufficient to induce wrinkling (Figure 7B).

Interestingly, in *Burkholderia pseudomallei *the lack rather than the overproduction of the Series A congener HHQ results in a wrinkly colony phenotype [61]. In addition, AQ signal overproduction has previously been shown to induce autolysis in *P. aeruginosa *populations, forming plaques that result in characteristic translucent zones in colonies [36], different from those we observed. Autolysis appears to be mediated by PQS rather than a Series A congener [65].

Taken together, our data can be rationalized as follows: In the wild-type, both Series A and Series B congeners are produced as LasR activates *pqsR *and *pqsH*. In the *lasR *mutant, Series A congeners accumulate and the Series A to Series B ratio increases because of (1) reduced *pqsH *expression and (2) presumably *lasR*-independent expression of *pqsR *[25] resulting in continued activation of *pqsA-E*. In this case, one or more Series A congeners activate Pel production postranscriptionally in a PqsR-independent fashion, manifesting in a wrinkled phenotype. Colony morphology would be affected by a combination of *pel*-dependent and independent mechanisms, as *lasR*-mediated wrinkling was only partially *pel*-dependent (Figure 3). The particular AQ compound could alter colony morphology by binding to a novel receptor protein or through membrane interactions. While both PQS and HHQ have been shown to associate with outer membrane LPS, only PQS induces vesicle formation [66]. Such distinct interactions might have direct macroscopic effects on colony morphology, but might also alter the periplasmic environment in a way that affects the signaling status of receptor proteins in the cytoplasmic membrane. Posttranscriptional regulation of Pel could be mediated via a transmembrane signaling pathway that involves the LadS/RetS/GacS/GacA two-component system, the RNA-binding protein RsmA and the small RNA RsmZ [67]. Pel translation has been shown to be repressed by the RNA-binding protein RsmA [68].

## Authors' contributions

MS and RG designed and RG performed the experiments. RG and MS analyzed and interpreted the results. RG drafted the manuscript and MS critically revised it. All authors read and approved the final manuscript.

## Supplementary Material

Additional file 1 **Table S1**. Oligonucleotides for deletion, overexpression, and reporter fusion constructs. Click here for file

Additional file 2 **Table S2**. List of insertion mutants with the location of the transposon insertion. Click here for file
