# Supplementary material for: Quorum sensing modulates colony morphology through alkyl quinolones in Pseudomonas aeruginosa
Source: BMC Microbiol. 2012 Mar 9;12:30. doi: 10.1186/1471-2180-12-30 (PMC3364869; doi:10.1186/1471-2180-12-30)
Supplement: Additional file 1 — Table S1. Oligonucleotides for deletion, overexpression, and reporter fusion constructs. [file 1471-2180-12-30-S1.PDF]

Table S1: Oligonucleotides for deletion, overexpression, and reporter fusion constructs.

Primer name	Forward/reverse	Primer sequence 5' to 3' [a]
pqsH deletion		
pqsH -del-1	Forward	N_6**CTGCAG**TTGACAGGAGCGGGGTC (*Pst* I)
pqsH -del-2	Reverse	ACTGGAAGGCATCGACATCAG
pqsH -del-3	Forward	CTGATGTCGATGCCTTCCAGTCCGAATGCCAGTCGCAGGC
pqsH -del-4	Reverse	N_6**AAGCTT**AGGACTTCAGCGCCAGTTGC (*Hin* dIII)
tpbA deletion		
PA3885-del-1	Forward	N_6**GAGCTC**GCGGCATCCCAGGACAATC (*Sac* I)
PA3885-del-2	Reverse	CAGGAAGGCGCCGAGCACG
PA3885-del-3	Forward	CGTGCTCGGCGCCTTCCTGCGCTTCGCCGTCTGCCATG
PA3885-del-4	Reverse	N_6**AAGCTT**AGTCGCCGACGAACAGGAT (*Hin* dIII)
pqsA-D constitutive expression		
pRG10-1	Forward	N_6**CTGCAG**TCTCCTGATCCGGATGCATATC (*Pst* I)
pRG10-2	Reverse	N_6**AAGCTT**CAGGCACAGGTCATCATCCAG (*Hin* dIII)
P_{pel} -*lacZ* -transcriptional fusion		
pRG11-1	Forward	N_6**AAGCTT**CCTCGGTGTGGCTGGTGCG (*Hin* dIII)
pRG11-2	Reverse	N_6**CTGCAG**ATGTTACGGCGGGACGGCAG (*Pst* I)

[a] Bold letters indicate the recognition sequence of the restriction enzyme in parentheses
