# Supplementary material for: Quorum sensing modulates colony morphology through alkyl quinolones in Pseudomonas aeruginosa
Source: BMC Microbiol. 2012 Mar 9;12:30. doi: 10.1186/1471-2180-12-30 (PMC3364869; doi:10.1186/1471-2180-12-30)
Supplement: Additional file 2 — Table S2. List of insertion mutants with the location of the transposon insertion. [file 1471-2180-12-30-S2.PDF]

Table S2: List of insertion mutants with the location of the transposon insertion

Candidate No. [a]	PA number	Gene	Gene function	Percent identity [c]	Location of insertion (bp)	Direction of insertion [e]
1	PA0396	*pilU*	Twitching motility protein PilU	100	634	R
2	PA0420	*bioA*	Adenosylmethionine-8-amino-7-oxononanoate aminotransferase	99.6	1160	R
3	PA0441	*dht*	Dihydropyrimidinase	100	451[d]	F
4	PA0500	*bioB*	Biotin syntahse	100	569	F
**5**	**PA0996**	***pqsA***	**Probable coenzyme A ligase**	**100**	**700**	**R**
**6**	**PA0996**	***pqsA***	**Probable coenzyme A ligase**	**100**	**801**	**F**
**7**	**PA0996**	***pqsA***	**Probable coenzyme A ligase**	**100**	**1211**	**R**
**8**	**PA0996**	***pqsA***	**Probable coenzyme A ligase**	**100**	**1294**	**R**
**9**	**PA0996**	***pqsA***	**Probable coenzyme A ligase**	**100**	**228**	**R**
**10**	**PA0998**	***pqsC***	**Beta-keto-acyl-acyl-carrier protein synthase**	**99.5**	**863**	**R**
**11**	**PA0998**	***pqsC***	**Beta-keto-acyl-acyl-carrier protein synthase**	**100**	**474**	**F**
**12**	**PA0998**	***pqsC***	**Beta-keto-acyl-acyl-carrier protein synthase**	**100**	**434**	**F**
**13**	**PA0999**	***pqsD***	**3-oxoacyl-[acyl-carrier-protein] synthase III**	**98.8**	**502**	**R**
**14**	**PA1003**	***pqsR***	**Transcriptional regulator PqsR, also known as MvfR**	**100**	**404**	**R**
15	PA1614	*gpsA*	Glycerol-3-phosphate dehydrogenase	98.2	326	R
16	PA3763	*purL*	Phosphoribosylformylglycinamidine synthase	100	2725	F
17	PA3763	*purL*	Phosphoribosylformylglycinamidine synthase	100	2725	F
18	PA4488	Hypothetical	Hypothetical protein	100	713	F
19	PA4760	*dnaJ*	DnaJ protein	100	175	R
20	PA4760	*dnaJ*	DnaJ protein	100	813	R
21	PA4762	*grpE*	Heat shock protein GrpE	100	259	R
22	PA5013	*ivlE*	Branched-chain amino acid transferase	100	81	R
23	PA5304	*dadA*	D-amino acid dehydrogenase, small subunit	100	712	F
24	N[b]	N[b]	N[b]	N[b]	N[b]	N[b]
25	N[b]	N[b]	N[b]	N[b]	N[b]	N[b]

[a] Bold represents candidates with an insertion in the *pqs* pathway

[b] N, no significant match found

[c] Percent identity of BLASTX match

[d] Insertion not in the gene but rather 451 bp upstream

[e] F, forward direction, R, reverse direction
